# Changes in Parent Psychological Flexibility after a One-Time Mindfulness-Based Intervention for Parents of Adolescents with Persistent Pain Conditions

**DOI:** 10.3390/children5090121

**Published:** 2018-09-03

**Authors:** Danielle Ruskin, Lauren Campbell, Jennifer Stinson, Sara Ahola Kohut

**Affiliations:** 1 Department of Anesthesia and Pain Medicine, The Hospital for Sick Children, Toronto, ON M5G 1X8, Canada; 2 Department of Psychology, The Hospital for Sick Children, Toronto, ON M5G 1X8, Canada; Lauren.campbell@sickkids.ca; 3 Department of Anesthesia and Pain Medicine and Research Institute, The Hospital for Sick Children, Toronto, ON M5G 1X8, Canada; Jennifer.stinson@sickkids.ca; 4 Lawrence S Bloomberg Faculty of Nursing, University of Toronto, Toronto, ON M5G 1X8, Canada; 5 Medical Psychiatry Alliance, The Hospital for Sick Children, Toronto, ON M5G 1X8, Canada; Sara.aholakohut@sickkids.ca; 6 Department of Psychiatry, University of Toronto, Toronto, ON M5G 1X8, Canada

**Keywords:** mindfulness, acceptance, psychological flexibility, parents, adolescence, chronic pain, irritable bowel disease, quantitative analysis, qualitative analysis

## Abstract

Parenting a child with chronic pain can be stressful and impact parent functioning in a variety of areas. Several studies have examined mindfulness-based interventions (MBIs) for parents of children with different health and mental health conditions. However, no studies to date have examined MBIs for parents of children with pain conditions. This study aimed to: (1) determine the feasibility and acceptability of a one-time MBI workshop for parents (*n* = 34) of adolescents with painful conditions (chronic pain and inflammatory bowel disease) who were participating in a concurrent mindfulness group for adolescents with pain, and (2) examine changes in parent mindfulness and psychological flexibility following the intervention. A mixed-method design was used. In terms of feasibility and acceptability, high recruitment and retention rates were observed, and parents reported high satisfaction scores with the workshop. Changes pre to post intervention showed that dimensions of parent psychological flexibility, but not parent mindfulness, improved following participation in the workshop. Qualitative analyses based on parent responses on a questionnaire uncovered seven themes of parent “takeaways” following participation in the workshop: Mindfulness Skills, Not Alone, Psychological Flexibility, Parent–Child Interactions, Self-Efficacy, Optimism/Positivity/Hope, and Awareness of Values. Taken together, these findings suggest that a one-time MBI workshop offered to parents whose teen was participating in a concurrent mindfulness group for pain is a feasible and promising intervention for parents of children with pain conditions.

## 1. Introduction

Chronic pain affects one-third of children and adolescents [1] and impacts children’s quality of life across multiple domains including physical (e.g., activity limitations, sleep disturbance), emotional (e.g., anxiety and depression), school attendance, and loss of social interactions [2,3]. Chronic pain is defined as pain that persists beyond expected healing time or recurrent pain occurring at least three times over a period of three months, including varying levels of disability [4,5,6].

Parenting a child or adolescent with chronic pain can be extremely stressful, sometimes devastating, and has been associated with far-reaching social, relational, and emotional challenges for parents [7]. For example, parents of children with chronic pain report higher levels of parent burden and emotional distress and a significant impact on marital relations, along with a sense that their family life is in a constant state of “limbo” [8]. Moreover, clinically significant levels of anxiety and depression have been documented in the parents of children with chronic pain [2]. Within this context, a renewed research focus on parents of children and adolescents with chronic pain is warranted, including a focus on determining optimal methods of intervening with parents either individually or as part of child-focused treatments [9]. 

Mindfulness-based interventions (MBIs) have emerged as a promising intervention for children and adolescents with chronic pain [10] given their versatility in targeting both physical and emotional distress [11]. Mindfulness has been defined as the nonjudgmental focus on and acceptance of present moment experiences [12]. A recent systematic review [13] assessed the efficacy of various psychological therapies delivered to parents of children with longstanding chronic illness (including cognitive behavioral therapy (CBT), family therapy, problem-solving therapy (PST) and multisystemic therapy), and showed some beneficial impact for CBT and PST on improving either parent or child outcomes, Yet, to our knowledge, no research to date has examined MBIs in the context of parenting an adolescent with chronic pain. Given that MBIs have been shown to improve anxiety and depression in adults [14], and that parent mindfulness has been associated with lower levels of parent “pain-promoting behaviors” in adolescents with chronic pain as well as improved adolescent functioning across social, emotional, and developmental domains [15,16], it follows that an MBI targeted to the parents of children with chronic pain may be particularly beneficial for both parents as well as their children.

Several studies have examined mindfulness groups for parents in the context of parenting. The majority of these studies have been on parents of children with developmental disabilities [17,18,19,20,21,22,23,24,25,26,27]. Parent mindfulness groups have also been studied for the parents of children with internalizing disorders [28,29], externalizing disorders [30,31,32,33,34,35], and mental health challenges more broadly [36,37]. In addition, one study has examined a mindfulness group for parents in the context of divorce [38]. In general, these studies have been experimental (i.e., randomized controlled trials) or quasi-experimental (i.e., pre–post) in design, consisted of mindfulness training ranging from two to 12 sessions, conducted as standalone parent interventions (i.e., without a concurrent child intervention), and did not adapt their mindfulness content to the child’s presenting problems.

Only one study to date has examined a parent mindfulness group for the parents of children with chronic health conditions [39]. In this study, an eight-week mindfulness-based stress reduction (MBSR) group was provided to caregivers of children with a variety of chronic health conditions (e.g., diabetes, asthma, epilepsy, Crohn’s disease, ulcerative colitis, irritable bowel syndrome, cancer). Prior to participation in the group, caregivers reported very high levels of stress and mood disturbance. Following the eight-week program, significant decreases in overall stress symptoms (32% reduction) and total mood disturbance (56% reduction) were reported. Of note, a limitation highlighted for this study was that the “active ingredients” driving the changes seen in the intervention were not studied. Based on previous research, two different components of mindfulness that warrant investigation as potential “active ingredients” of mindfulness training are attention and awareness of what occurs in the present moment [40] and psychological flexibility [16].

Another study investigated the impact of an eight-week Acceptance and Commitment Therapy (ACT) group for parents of children with chronic pain, and showed improvements in psychological flexibility post intervention (i.e., parents’ abilities to accept their distress about their child’s suffering, attend to the present moment, and focus on broader goals rather than be distracted by worries or thoughts) [41]. While mindfulness strategies are used in ACT, the ACT approach differs from mindfulness in that ACT focuses on commitment to behavioral change to realize valued life goals, whereas mindfulness involves focusing attention on the present moment nonjudgmentally. To our knowledge, no studies to date have examined a parent mindfulness group for the parents of children with pain conditions.

Given the dearth of research on mindfulness interventions for parents of adolescents with chronic pain conditions and the stressful impact that parenting an adolescent with chronic pain can have, the primary aim of this study was to develop and pilot a one-time mindfulness workshop for parents of adolescents with pain conditions. The decision to offer a one-time (versus multi-session) workshop was based on feedback obtained from parents in the context of clinic care: it is difficult for parents to commit to regular attendance at a weekly group. In addition, as some clinical settings may lack the resources to conduct a multi-week parent group (i.e., two facilitators to staff the parent group and another two to staff the teen group, should the teen group be offered), it was of interest to explore whether a single session offering would be acceptable to parents and whether it would show any impact on parent outcomes.

The primary aim of the present study was to determine the feasibility and acceptability of a one-time mindfulness group intervention program for parenting adolescents with painful conditions (complex chronic pain conditions, inflammatory bowel disease (IBD)) within the context of a concurrent mindfulness group within which their child was participating. The second aim was to examine the preliminary impact of the intervention on parent mindfulness and parent psychological flexibility. With respect to the second aim, it was hypothesized that parent scores on mindfulness and overall psychological flexibility would increase following participation in the group.

A third exploratory goal was to examine whether the mindfulness group had a differential impact on the two outcome variables of interest (mindfulness and psychological flexibility) depending on the child’s health condition (complex chronic pain condition versus inflammatory bowel disease). The assessment of workshop impact based on the child’s health condition was selected as an aim given that these two conditions differ in disease course, and thus mindfulness content may be received differently (chronic pain is a persistent condition often without an identifiable organic cause for the pain, while IBD is a relapsing/remitting condition with an organically identifiable source of pain). Additionally, these conditions have been shown to have differential impact on areas known to be affected by MBIs such as pain catastrophizing, functional impairment, and mood/anxiety [42]. A fourth exploratory goal was to qualitatively examine parents’ experiences of the workshop with a particular focus on their main take-home messages.

## 2. Methods

### 2.1. Study Design

A mixed-method design was used employing quantitative data on levels of mindfulness and parent psychological flexibility administered pre and post participation in the parent workshop along with qualitative data from open-ended questionnaire items administered immediately after the parent workshop. The parent mindfulness workshop was provided to parents of adolescents who were participating in the MBI-Adapted for chronic pain (MBI-A), an eight-week mindfulness group adapted for children with a chronic health condition (see Ruskin et al., 2017 [10] for further description of group content and structure). For the current study, we combined data from parents whose child either participated in the MBI-A group for adolescents with chronic pain, or the MBI-A group for adolescents with IBD. Content of the parent workshop was identical regardless of whether parents had a teen in the chronic pain or the IBD group. The first parent workshop was provided in the spring of 2015 to parents of children with chronic pain (*n* = 11); the second parent workshop was provided in the fall of 2016 to parents of children with IBD (*n* = 20), and the third parent workshop was provided in the fall of 2017 to parents of children with chronic pain (*n* = 3). 

Baseline questionnaires (mindfulness and psychological flexibility—see measures below) were administered one week prior to the workshop and were distributed via email using the Research Electronic Data Capture (REDCap) platform [43]. These questionnaires were administered again immediately following the workshop along with a post workshop questionnaire.

### 2.2. Participant Recruitment Procedure

Parents of participants were recruited from a major pediatric tertiary care hospital in Canada. Ethics approval was obtained from the Hospital for Sick Children’s Research Ethics Board (No. 1000045165, approved 4 June 2014 for Chronic Pain; No. 1000048956, approved 14 July 2015 for IBD). Parents of children with chronic pain were recruited from this hospital’s chronic pain clinic, while parents of children with IBD were recruited from this hospital’s IBD clinic. Parents were deemed eligible if they had a child who was participating in one of the mindfulness groups delivered either to adolescents with chronic pain or adolescents with IBD. Adolescent patients in the mindfulness group were eligible for the study if they were between the ages of 12–18, and were diagnosed with a chronic pain condition (for the chronic pain MBI-A group) or with Crohn’s disease or ulcerative colitis (for the IBD MBI-A group). Patients were excluded from participating in the study if they had a severe cognitive impairment that would impede their ability to participate in the mindfulness group, as per their treating health care provider.

Parents were approached to participate immediately following their child’s scheduled clinic appointment. Eligible parents were provided a letter outlining the purposes of the study and nature of the MBI-A program. All of the parents were either met in person or contacted by phone by the Clinical Research Project Coordinator (CRPC) or the Clinical Research Project Assistant (CRPA) to share additional information about the study. Parents were told that their participation in the parent workshop was optional/voluntary. Informed, written consent was obtained from all of the participants.

### 2.3. Intervention

The parent mindfulness workshop was provided concurrently with the second session of the eight-session MBI-A within which their adolescent was participating. Parents met in a separate room from their teens. The workshop was 2 h in duration. All of the workshops were identical in content and were guided by a structured schedule and a set of pre-determined mindfulness activities (see Table 1). Throughout all of the exercises within the workshop, from the icebreaker at the beginning of the session to each of the activities within the session, participants were encouraged to use mindful awareness during the activity (i.e., noticing their thoughts, emotions, physical sensations, judgments). The same facilitator (D.R.) facilitated each workshop, lending to consistency across the administration of the workshops.

Treatment fidelity was ensured through the use of a semi-structured session guide that was implemented consistently for both MBI-A group programs. Further, the same facilitator was responsible for leading all of the teen groups, limiting the variability of intervention delivery between groups.

### 2.4. Data Collection

#### 2.4.1. Demographic Information

Demographic information was captured using the Adolescent Health Information Form (AHIF), which was developed for a previous study [10]. The AHIF was administered to gather the following demographic and health information: sex, adolescent age, diagnosis, and duration of pain. Disease activity was measured using the Self-Reported Disease Activity Questionnaire for IBD. This tool is modified from the Pediatric Ulcerative Colitis Activity Index (PUCAI) for ulcerative colitis [44], and weighted Pediatric Crohn’s Disease Activity Index (PCDAI) for Crohn’s disease [45].

#### 2.4.2. Mindful Attention Awareness Scale (MAAS)

Mindfulness was assessed using the Mindful Attention Awareness Scale (MAAS) [40]. This 15-item instrument measures the tendency to be attentive to and aware of moment-to-moment experiences in daily life. Items are rated on a Likert scale ranging from 1 (almost always) to 6 (almost never), with higher total scores indicating higher levels of mindfulness. The instrument focuses on the presence or absence of attention and awareness of what occurs in the present. Items include, “I find myself doing things without paying attention”, and “I break or spill things because of carelessness, not paying attention, or thinking of something else”. The MAAS has reported internal consistency (coefficient alpha = 0.82) and expected convergent and discriminant validity correlations.

#### 2.4.3. Parent Psychological Flexibility Questionnaire (PPFQ)

The Parent Psychological Flexibility Questionnaire (PPFQ) was developed to measure parental psychological flexibility in the context of pediatric chronic pain [15]. The PPFQ is a 31-item measure of parent capacity to accept their own distress about their adolescent’s pain, attend to the present moment, and focus on broader goals and values rather than being distracted by worry, unpleasant feelings, or difficult thoughts. Items were developed to reflect acceptance, cognitive diffusion, values-based action, and mindfulness [41]. Parents rate each item on a Likert scale from 0 (never true) to 6 (always true). Examples of items are “Even though my child has pain, we can continue to do things that are important and enjoyable”, and “It is ok for my child to experience pain”. For the present study, all 31 items were administered, and a total overall score and four subscales were calculated based on a recent factor analysis which refined the scale to 17 items [16]. These subscales included values-based action (five items), pain acceptance (four items), emotional acceptance (five items), and pain willingness (three items). Higher scores indicate greater psychological flexibility. The total scale has good internal consistency (α < 0.87), and the three larger subscales have demonstrated acceptable to good internal consistency (α between 0.74–0.88); however, the three-item subscale (pain willingness) has shown marginal internal consistency (α = 0.67 for mothers and 0.60 for fathers) [16].

#### 2.4.4. Post Session Questionnaire

The post session questionnaire was a measure developed for this study to obtain feedback from participants regarding their experience in the workshop. A single item asked participants to rate their “overall satisfaction with today’s workshop”. Ratings were made from 0 (“not at all satisfied”) to 10 (“the most satisfied ever). In addition, parents were asked to rate their “ability to model being mindful to their children” (on a scale of 1 to 10) both before and after the group. Participants were also asked to indicate three take-home lessons they learned from the group.

### 2.5. Data Analysis

#### 2.5.1. Descriptive Analyses

Chi-squared and *t*-test demographic analyses were run to examine potential differences between the chronic pain and IBD groups in terms of parent and adolescent gender, and adolescent age.

#### 2.5.2. Quantitative Analyses

*Primary Outcomes.* Recruitment rates and percent of completion of outcome measures were used to examine participant recruitment and retention (feasibility). Calculation of the mean was used to examine parents’ average satisfaction rating, and thus was a measure of treatment acceptability.

*Secondary Outcomes.* To compare participants (pre versus post) on the secondary outcome variables (mindfulness and psychological flexibility), two two-way repeated measures analyses of variance (ANOVAs) were planned and conducted with time (pre versus post) and adolescent pain diagnosis (chronic pain versus IBD) as between group factors. A paired-samples *t*-test was used to compare parents’ scores (pre versus post workshop) on their ability to model being mindful to their children. This was an item on the post session questionnaire.

#### 2.5.3. Qualitative Analyses

In order to analyze the data qualitatively, an inductive qualitative simple content analysis was employed [46,47]. This approach allows for a systematic classification of the data into categories based on patterns. Since a coding schema is not imposed, this approach allows for novel insights and understanding from the participants’ perspective, which was grounded in their experiences [46]. Parents’ written responses on the satisfaction questionnaires were reviewed by the two coders (D.R., L.C.). A preliminary coding approach was developed through independent review by both coders, identifying themes and patterns, followed by a consensus meeting where the coding approach was refined in order to best capture the data. Broad themes as well as subthemes were coded. Both coders coded 100% of all of the written responses. Inter-rater reliability between the two coders was 93% for the coding of broad themes. Coders met again to agree on the code to assign in instances where there was a disagreement.

## 3. Results

### 3.1. Demographics

A total of 34 parents participated across the three groups. Demographic characteristics are presented in Table 2. Across the three groups, there were four occasions when both parents of an adolescent participated in the group (one occasion in the first chronic pain group, three occasions in the IBD group). Adolescents in the chronic pain group were significantly older (M = 15.54, SD = 1.56) than adolescents in the IBD group (M = 14.17, SD = 1.19). In terms of adolescent gender, there was a significant association between adolescent gender and adolescent diagnosis (i.e., chronic pain versus IBD) (χ^2^(1, *N* = 34) = 11.38, *p* = 0.001), such that there were more males in the MBI-A group for adolescents with IBD versus chronic pain. In terms of parent gender, there was not a significant association between parent gender and adolescent diagnosis (χ^2^(2, *N* = 34) = 1.58, *p* = 0.453). Forty percent of adolescents in the IBD group (8/20) had scores of less than 10 on the Self-Reported Disease Activity Questionnaire for IBD (see Table 2), indicating they were in remission at the time of the parent group.

### 3.2. Primary Outcomes

#### 3.2.1. Recruitment and Retention (Feasibility)

Spanning across all three groups, 94% of parents (i.e., 32/34) who were offered the group participated in the group. The first chronic pain group was offered to 11 parents, all of whom (100%) agreed to participate. The second chronic pain group was offered to five parents, four of whom (80%) agreed to participate. The IBD group was offered to 21 parents, 20 of whom (95%) agreed to participate. There was one occasion across all three groups when a parent left the group at the beginning of the workshop because her daughter was sick. In terms of completion of outcome measures, 100% of parents completed the pre-group measures and 82% of parents completed all of the post-group measures. In terms of completion of post-group measures, rates of completion were higher when participants completed the measures electronically (91% completion rate) compared to using paper-based forms (63% completion rate).

#### 3.2.2. Treatment Acceptability

Spanning across all three groups, the parents’ average satisfaction rating of the workshop was 8.12/10 (range = 6 to 10, SD = 1.17). Independent samples *t*-test indicated that there was no significant difference in satisfaction ratings between parents of children with chronic pain (M = 8.25, SD = 1.27) and parents of children with IBD (M = 8.05, SD = 1.15); t(28) = 0.434, *p* = 0.453. Parents were asked to rate their ability to model being mindful to their children (on a scale of 1–10) both before and after the group. Independent samples t-tests revealed that there was not a significant difference across groups in parent report on this ability prior to the group [(chronic pain: M = 4.80, SD = 2.78); (IBD: M = 4.35, SD = 2.01); t(28) = 0.509, *p* = 0.615) or following the group [(chronic pain: M = 6.50, SD = 2.37); (IBD: M = 6.65, SD = 1.63); t(28) = 0.204, *p* = 0.093). Grouping the parents as a whole, a paired-samples *t*-test indicated that parents reported significantly higher perceptions of their ability to model being mindful to their children after the group (M = 6.60, SD = 1.87) compared to before (M = 4.50, SD = 2.26), t(29) = −7.58, *p* < 0.001).

### 3.3. Secondary Outcomes

#### 3.3.1. Mindfulness

Using the MAAS as the dependent variable, a two-way repeated measures analysis of variance (ANOVA) was conducted with time (pre versus post) and adolescent pain diagnosis (chronic pain versus IBD) as between-group factors. The main effects for time and adolescent pain diagnosis were not significant (F(1,26) = 0.09, *p* = 0.763; F(1,26) = 0.61, *p* = 0.442; respectively), and neither was the two-way interaction (F(1,26) = 0.37, *p* = 0.550).

#### 3.3.2. Psychological Flexibility

Using the PPFQ total score as a dependent variable, one two-way repeated measures analysis of variance (ANOVA) was conducted with time (pre versus post) and adolescent pain diagnosis (chronic pain versus IBD) as between group factors.

*Total flexibility.* For total psychological flexibility, there was a significant main effect of time (F(1,26) = 5.18, *p* = 0.031), indicating that parent overall psychological flexibility was higher following the workshop (M = 47.19, SD = 12.18) than before the workshop (M = 44.14, SD = 13.20). There was no significant effect of adolescent pain diagnosis (F(1,26) = 0.25, *p* = 0.622) nor two-way interaction (F(1,26) = 0.09, *p* = 0.764).

#### 3.3.3. Post Hoc Exploratory Analyses

After observing that overall psychological flexibility was significantly higher following the workshop, exploratory analyses were completed to determine whether the scores on each of the four subscales of psychological flexibility (i.e., values-based action, emotional acceptance, pain acceptance, and pain willingness) might change following the workshop. 

*Values-Based Action.* For the values-based action subscale, there was a significant main effect of time (F(1,26) = 4.66, *p* = 0.040), indicating that parent values-based action was higher following the workshop (M = 47.84, SD = 10.70) than before the workshop ((M = 45.24, SD = 11.26). There was no significant main effect of adolescent pain diagnosis (F(1,26) = 0.11, *p* = 0.740) nor two-way interaction (F(1,26) = 0.08, *p* = 0.775).

*Emotional Acceptance.* For the emotional acceptance subscale, there was no significant main effect of time, adolescent pain diagnosis, nor two-way interaction (F(1,26) = 1.33, *p* = 0.260; F(1,26) = 1.75, *p* = 0.197; F(1,26) = 0.296, *p* = 0.591, respectively).

*Pain Acceptance.* For the pain acceptance subscale, the main effect of time (i.e., impact of the workshop) was approaching significance (F(1,26) = 3.15, *p* = 0.088). There was no significant main effect of adolescent pain diagnosis nor two-way interaction (F(1,26) = 0.57, *p* = 0.458; F(1,26) = 1.07, *p* = 0.311, respectively).

**Figure 1:**
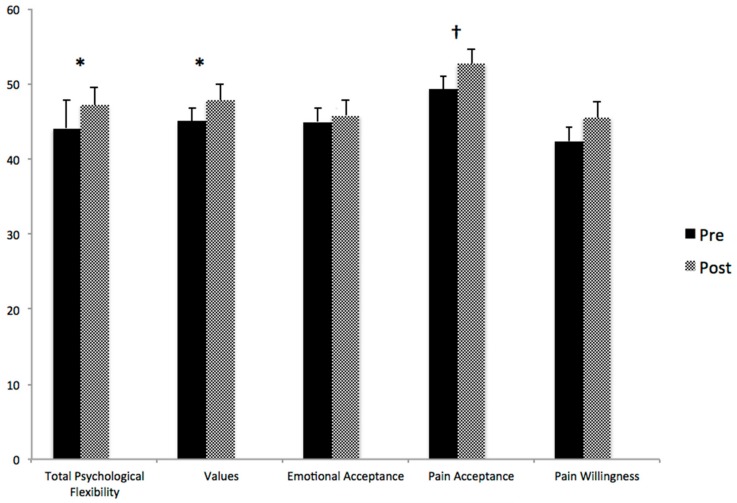
Parent Psychological Flexibility Questionnaire (PPFQ) scores pre and post workshop. * *p* < 0.05, ^†^ *p* < 0.10.

*Pain Willingness.* For the pain willingness subscale, there was no significant main effect of time, adolescent pain diagnosis, nor two-way interaction (F(1,26) = 1.67, *p* = 0.208; F(1,26) = 0.125, *p* = 0.727; F(1,26) = 0.773, *p* = 0.387, respectively). Figure 1 represents PPFQ Total Score and Subscale Scores pre and post workshop.

### 3.4. Qualitative Analysis (Post-Session Questionnaire)

**Figure 2:**
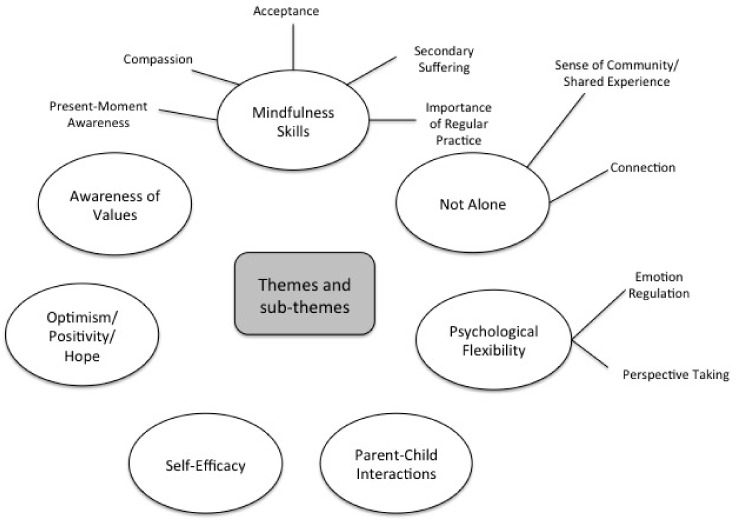
Themes and subthemes resulting from qualitative analysis of the post session questionnaire.

Qualitative analysis revealed seven main themes: Mindfulness Skills, Not Alone, Psychological Flexibility, Parent–Child Interactions, Self-Efficacy, Optimism/Hope, and Awareness of Values. Some of these themes include multiple subcategories that reflect a variety of ideas within the primary theme (Figure 2). Table 3 provides additional illustrative quotations for each theme and subtheme. No negative experiences associated with the workshop were reported.

#### 3.4.1. Mindfulness Skills

Parents articulated multiple ways that the Parent Mindfulness Workshop helped them to develop mindfulness skills including: being more present in the moment, practicing compassion, accepting their child’s condition, recognizing the secondary distress that can occur due to their own thoughts and emotions about their child’s pain, and integrating mindfulness practice into their daily lives. Each of these subcategories will be described below.

*Present-Moment Awareness.* Parents described the importance of bringing their attention to the present moment in relation to their experience of parenting a child with a health condition. As one participant stated, “trying to be more present and not just making it through the day” (Participant, IBD Group). Parents also commented on how present moment awareness can assist in reducing distress “to be at ease… how to “be” and “stay quiet”… or try at least a little harder to be that way”. (Participant, Chronic Pain Group). Purposefully bringing attention to the here and now also was seen as assisting in noticing what really matters “to take the time to really realize what is important in the moment” (Participant, IBD Group).

*Compassion.* Parents commented on increased awareness of the importance of compassion toward themselves and others. Several parents remarked that they left the workshop with an increased sense of compassion toward their spouses—“I need to support my partner’s feelings more” (Participant, IBD Group)—and also toward their child—“... how to be more supportive of my child and her chronic pain” (Participant, Chronic Pain Group). In addition, parents recognized the importance of taking time for themselves: “… as parents we need to work at finding ‘breaks’ from our focus on our daughters. Mindfulness is a tool to step away mentally from our suffering to be kind to ourselves” (Participant, Chronic Pain Group). They also remarked on the value of avoiding harsh judgments: “Knowing that I am not perfect in this journey is okay” (Participant, Chronic Pain Group).

*Acceptance.* Parents described awareness of the importance of accepting their child’s condition and moving forward. One parent described her take-home lesson as the following: “We may not be able to cure the pain, but can learn to live with it and still enjoy life” (Participant, Chronic Pain Group). Another parent noted the importance of: “... not dwelling on my child’s pain. For her sake, not talking about pain (unless my child chooses to) can help with pain management” (Participant, Chronic Pain Group). Finally, one parent commented: “pain is not a bad thing” (Participant, Chronic Pain Group).

*Secondary Suffering/Distress.* Parents described several take-home lessons regarding an awareness of how they are not only coping with their child’s health condition, but also the secondary distress that comes with ruminative thoughts and difficult emotions about their child’s health condition, which is termed secondary suffering. One parent commented on developing more awareness of secondary suffering: “Through the ‘water in the cup’ exercise, I realized how much I’m still consumed by my child’s medical/pain needs.” (Participant, Chronic Pain Group). Another commented that they were: “... understanding the potential for how much of my thinking can be consumed by worry for my child (Participant, IBD Group). Some parents noted take-home messages regarding the importance of ways to reduce secondary suffering, such as “to try not to manifest my emotions onto my body—to relax my physical body” (Participant, IBD Group), while another commented, “I can be much stronger when I don’t think too long or too hard about myself” (Participant, IBD Group). Another parent noted an intention to “let thoughts be thoughts and not let them take over” (Participant, IBD Group).

*Importance of Regular Practice.* The importance of engaging in a regular mindfulness practice was highlighted as a take-home lesson by several parents. For example, one parent commented that they will begin to integrate mindfulness “in everyday life situations” (Participant, Chronic Pain Group). Another parent remarked that “mindfulness is a practice I should do more actively and check in with my child on” (Participant, IBD Group).

#### 3.4.2. Not Alone

Parents described how the workshop provided them with a sense of feeling “not alone”. Some parents reported their sense of feeling not alone very explicitly: “I am not alone” (Participant, IBD), “I’m not the only parent who suffers” (Participant, Chronic Pain Group), and “we aren’t alone—many people/families are dealing with pain issues… there are people and resources available to help” (Participant, Chronic Pain Group).

Within this broad theme of feeling not alone, two subthemes emerged: experiencing a sense of community/shared experience, and feeling a sense of connection with other parents going through this shared experience. Each of these subthemes will be described below.

*Sense of Community/Shared Experience.* Parents noted that being in the group fostered a sense of community and of a shared experience with others faced with similar situations. For example, one parent commented that an important take-home message from the group was “knowing that there are other parents with children going through the same thing” (Participant, Chronic Pain Group). Other parents reported a feeling of commonality with other parents: “others feel the same way, so we are not alone” (Participant, IBD Group), and “our struggles in parenting a child in pain are similar” (Participant, Chronic Pain Group). Finally, one parent took away that: “there are support systems to help in hard times” (Participant, Chronic Pain Group).

*Connection.* Parents also remarked on coming away from the group with a sense of connection with other parents. One parent noted: “It was helpful to connect with a community of other parents and to hear their concerns and thoughts” (Participant, Chronic Pain Group). Another parent spoke of the benefit of connection through listening: “[It was a] good experience to listen to other parents’ comments and struggles” (Participant, IBD Group), “I really enjoyed meeting the staff and parents and listening to all their experiences” (Participant, IBD Group). Finally, parents highlighted their sense of connection through speaking and sharing openly: “[It was good to] talk openly about our children, their pain, and experiences” (Participant, IBD); “parent sharing was very helpful” (Participant, IBD Group).

#### 3.4.3. Psychological Flexibility

*Emotion Regulation.* Parents described take-home messages of developing more flexibility in how they respond to challenging situations with their teens. Several parents described learning practices to respond rather than react to their experiences. As one parent noted, it was helpful to learn “techniques for being mindful and controlling my temper in volatile situations” (Participant, IBD) while another appreciated “taking a step back from reacting with emotion” (Participant, IBD). Many parents liked the STOP technique that they practiced during the workshop that can help avoid reacting in knee-jerk ways to difficult situations. As one parent commented: “I learned to use STOP as a way to allow myself to process and respond to situations” (Participant, Chronic Pain Group). Another parent remarked “it was good to learn to stop, take a moment, observe, and proceed” (Participant, Chronic Pain Group).

*Perspective Taking.* The ability to consider other perspectives and to widen the lens within which they view their situations was described as helpful by some participants. For example, one parent after participating in the group commented “how grateful I am that we are probably past the worst of it and that we can get back to normal” (Participant, IBD Group), and another parent commented that “others truly have it harder than our family” (Participant, Chronic Pain Group). Another parent noted the impact of having a more flexible perspective with respect to her child’s condition: “perhaps there are positives to be taken out of my son’s illness” (Participant, IBD Group).

#### 3.4.4. Parent–Child Interactions

Participation in the group facilitated parent awareness of the importance of their interactions with their children, and more specifically, the impact of parent responding on child well-being. Parents remarked that the group helped them better understand “how my actions can impact my daughter’s health” (Participant, IBD) and “how to be more supportive of my child and her chronic pain” (Participant, Chronic Pain Group). Other parents indicated that they hoped to achieve a collaborative mindfulness practice with their children: “Mindfulness is a practice I should do more actively and check in with my child on” (Participant, IBD), and, “We need to work collectively to develop and maintain coping strategies and work with our daughter to achieve some level of normalcy” (Participant, Chronic Pain Group). Finally, one parent spoke of the importance of parent self-care in terms of the impact that this will have on child wellness: “I need to learn how to take care of myself so I can look after my child” (Participant, Chronic Pain Group).

#### 3.4.5. Self-Efficacy

Other parents felt that participation in the group engendered a greater sense of parent self-efficacy regarding their mindfulness practice: “I realized that I have been improving some mindfulness tactics over the past year” (Participant, Chronic Pain Group), and “I am a lot further along the mindfulness path than I thought I was; I think I have been trending in that direction for a while” (Participant, IBD Group). Under the umbrella of parent self-efficacy, some parents shared positive self-affirmations: “I am not doing a bad job” (Participant, IBD Group) and “I’m on the right track” (Participant, IBD Group).

#### 3.4.6. Optimism/Positivity/Hope

Some parents reported a renewed sense of optimism, positivity, and hope following participation in the group. An emphasis on gratitude was a key takeaway for some parents: “gratitude that my daughter is taking part in the group and having the opportunity to learn skills to help her manage her pain” (Participant, Chronic Pain Group). In a similar vein, some parents remarked on their desire to choose positivity as their mental framework moving forward: “My son will come out with a positive experience” (Participant, IBD Group), and “Perhaps there are positives to be taken out of my son’s illness” (Participant, IBD Group). Another parent demonstrated a greater awareness of the emotional benefits of choosing to focus on the positive: “... the joy that thinking about positive experience brings” (Participant, IBD Group).

#### 3.4.7. Awareness of Values

Exercises during the workshop of helping parents identify their parenting values and respond to their children accordingly were described as helpful. For example, one parent noted “the ‘5 yrs’ from now exercise really helped bring clarity to what I feel is important to ensure my daughter’s happiness and knowing how I should behave accordingly” (Participant, IBD Group). Another parent indicated that it was helpful to “isolate the one value that I want to teach my children before they become adults so I don’t look back and find I was so caught up” (Participant, IBD Group). Finally, one parent noted a desire to abide by her values on a daily basis for her daughter: “awareness of the values I want to uphold for her every day” (Participant, Chronic Pain Group).

## 4. Discussion

To our knowledge, this is the first study of its kind to examine a one-time mindfulness workshop for parents of adolescents with pain conditions. Accordingly, the present pilot study provides novel contributions to the field on mindfulness-based interventions delivered to parents. A notable strength of this study was the adaptation of the workshop’s mindfulness content to the issues that parents experience in the context of their adolescents’ pain condition.

The primary aim of this study was to develop and pilot a one-time mindfulness workshop for parents of adolescents with pain conditions and assess the feasibility and acceptability of this group. In terms of feasibility, the high recruitment and retention rates (95% and 82–100%, respectively) suggest that this one-time workshop is an offering that parents are likely to attend and provide feedback on. A contributing factor to the high recruitment rate may be because the parent mindfulness workshop was intentionally held at the same time as the concurrent group within which their teen participated, thus facilitating parent attendance. Given the stress and demands associated with parenting a child with chronic pain [7], the decision to hold the parent workshop at the same time as the teen group was purposeful, as many parents may not have had the extra time or means to come to the hospital solely for their own involvement. The one-time nature of the workshop (versus asking parents to attend an eight-week group, which would have been concurrent to the eight-week teen group) likely also contributed to the high recruitment and retention rates. Regarding the retention rates, it is notable that while retention rates (i.e., completion of outcome measures) were high overall, completion of study questionnaires (completed immediately post workshop) was higher when parents used electronic measures compared to paper-based forms (i.e., 91% compared to 63%). Future researchers may wish to bear this in mind when designing similar studies. In terms of acceptability, parents reported high satisfaction scores with the group (8.2/10), and findings showed that the workshop improved parents’ ability to model being mindful to their teen.

The second aim of this pilot study was to examine possible changes in parent mindfulness and parent psychological flexibility following the intervention. In line with hypotheses, dimensions of parent psychological flexibility increased following participation in the workshop. Specifically, improvements were seen post workshop in parents’ capacity to focus on their broader goals and values, even while their child experiences pain (e.g., “even though my child has pain, we can do things that are important and enjoyable”). In addition, parents showed reductions in the emphasis that they placed on stopping or controlling their adolescent’s pain (e.g., “when my child is in pain, the most important goal is to make it stop”). These findings are consistent with those of Wallace et al. [41], who observed an increase in parent psychological flexibility following an eight-week ACT group for parents of children with chronic pain. As aforementioned, mindfulness strategies are used in ACT. However, the two approaches differ in regards to their primary areas of focus (e.g., ACT places emphasis on commitment to behavioral change to realize valued life goals, whereas mindfulness places its focus on the nonjudgmental awareness and acceptance of present moment experiences). Thus, while results from the current study provide complementary findings to those of Wallace et al. [41], our results are also novel in that they pertain to an increase in parent psychological flexibility following a workshop focused on an introduction to mindfulness skills. In addition, findings from the current study provide initial evidence that it is possible to improve parent psychological flexibility even after a one-time workshop. Although this finding is preliminary, it is encouraging for those who may not have the resources or setting to provide multi-week mindfulness interventions.

It is possible that an immediate impact of the workshop was seen because workshop content was specifically adapted to pain and pain acceptance. This finding adds credence to our rationale for tailoring the workshop content so that it was relevant and specific to parents of children with painful conditions rather than teaching generic mindfulness principles. Indeed, there is a growing body of research showing that tailoring an intervention message to an individual’s specific circumstance, including aspects of their health condition, can result in better outcomes [48]. The improvements observed in parent psychological flexibility are encouraging, given that this construct has been associated with adaptive parenting behavior in non-clinical samples [49,50], and may moderate the relationship between parent and child distress [50]. Furthermore, research on parents of children with chronic pain has shown that parent psychological flexibility is positively associated with the level of functioning in the child [15,16].

Counter to hypotheses, parent mindfulness did not increase following participation in the workshop. At first glance, this finding was surprising, given that a core aspect of workshop content was focused on mindfulness principles. However, several studies on mindfulness (adolescents and adults) have shown that mindfulness skills increase with practice over time [51,52,53], so post-treatment improvements are often seen months following the intervention rather than immediately afterwards. Therefore, in the current study, the lack of improvements observed in mindfulness skills may be due to our measurement of this construct immediately after the workshop. Future studies should consider assessing mindfulness at an additional later time point (e.g., three months post workshop) once participants have had the opportunity for practice.

Of interest in this study was whether parents of children with chronic pain might experience the workshop differently than parents of children with IBD, given that these conditions have different characteristics. For instance, while both conditions involve pain/discomfort, IBD is a relapsing and remitting disease (indeed, 40% of the parents of adolescents with IBD had a teen who was in remission), while chronic pain is a persistent condition (teens with chronic pain had been living with persistent pain for, on average, five years). While differences between diagnostic groups were not seen on quantitative outcomes, slightly different themes emerged between these two diagnostic groups in the take-home messages parents articulated. For example, parents of teens with chronic pain commented on the importance of workshop material that assisted them in accepting their child’s condition, which may be relevant given the chronicity of their teen’s condition. Parents of adolescents with IBD spoke of broadening their perspectives to feel grateful for getting back to normalcy and being able to take positives from their child’s condition. Rolland [54] has written extensively on how the characteristics of children’s health conditions can differentially impact the family system. According to Rolland [54], relapsing/remitting conditions with periods of normalcy and periods of illness (e.g., as in IBD) can lead to strain in families that is related to the uncertainty on when recurrence will occur, and requires family flexibility in moving back and forth between periods of normalcy and illness. In contrast, chronic conditions (e.g., chronic pain) may be more predictable, but can be exhausting for parents given their chronicity. While this study’s small sample size precluded an in-depth investigation of diagnostic differences between parents of children with IBD and parents of children with chronic pain, other studies may wish to consider assessing differences on the impacts of MBIs delivered to parents based on factors such as the course of their child’s condition (chronic, progressive, or relapsing/remitting) or time phases of the child’s condition (e.g., crisis/pre-diagnostic phase, chronic, terminal).

Given this discussion, a question that emerges is whether it makes sense to offer the parent workshop to parents regardless of their child’s condition (i.e., combine parents into one workshop) or whether parents should be grouped with other parents who have a child with a similar condition, as we did in this study. While the current study’s data is not sufficient to answer this question, several considerations are offered. First, if staffing or resources do not allow for running multiple workshops for different conditions, results from the current study argue to at minimum provide the workshop to parents regardless of child diagnosis, given that the content was generally helpful for all of the parents (i.e., improvements in psychological flexibility and parents’ ability to model being mindful to their children were seen regardless of child diagnosis). However, if not constrained by limitations due to staffing or resources, it should be noted that one of the most popular take-home messages from the workshop articulated by parents was having a “shared parenting experience” of connecting with parents who had a child with the same diagnosis. As one parent stated: “I am not the only parent that is going through a difficult situation with a child that suffers from chronic pain.” One risk of combining groups is for parents who have children with a chronic disease to feel even more alone when hearing reflections of parents who have children with periods of disease remission. Ultimately, the most reasonable next step is to ask parents their preference: would they prefer being with other parents who have a child with a similar condition, or are they open to attending with other parents who have a child with a health condition that might be different than that of their child’s? In our research program, teens overwhelmingly provided unsolicited feedback of wanting to participate in MBI groups with teens who also had their diagnosis. This feedback led to the development and implementation of an MBI group specifically for adolescents with chronic pain, which many adolescents articulated was a key benefit of their group experience [11].

Taken together, the results of the current pilot study provide initial support for the efficacy of a one-time mindfulness workshop in improving psychological flexibility for the parents of children with pain conditions. Our findings add to those of previous studies supporting the use of parent mindfulness in the context of other child and pediatric conditions (e.g., chronic health conditions, developmental disabilities, mental health challenges, etc.), and indicate that an important takeaway from the group was parents feeling “less alone”. This study provides preliminary evidence that a single workshop can be cost-effective and efficient in improving clinically relevant outcomes related to parenting a child with a pain condition.

### 4.1. Limitations

When interpreting study findings, several limitations should be considered. First, post workshop measurements were obtained directly following the workshop, which may not have provided parents sufficient time to practice the mindfulness skills. Second, it is possible that qualitative differences in the “takeaway” messages across diagnostic groups may have been a product of “group think” (i.e., shaping of group takeaways based on lines of commentary and parents’ interactions and loss of individual opinions) rather than actual differences due to the nature of the teen’s diagnosis. Third, parents who chose to participate in this study (participation was voluntary) may have been particularly motivated to learn mindfulness strategies given that their children were attending the mindfulness group for teens (MBI-A). Thus, the generalizability of the present study’s findings in a less motivated population is unclear. Fourth, we do not know if the effects on psychological flexibility are maintained over time. Thus, inclusion of a follow-up point three months following the workshop is recommended. Finally, we did not obtain data on the adolescent’s level of pain. However, previous research has shown that children’s pain ratings are not a strong predictor of long-term outcomes [55], and thus may not be a crucial variable.

### 4.2. Future Directions

Future studies may wish to collect data on parent mindfulness and psychological flexibility post workshop at a more distal time point, (e.g., three months post workshop) to allow time for parents to practice skills. On a related note, it is advised to collect data on the amount and extent of parent practice to determine whether the amount of practice may moderate outcomes, especially given that the practice of skills has been related to outcomes in other MBI studies [56,57]. Other directions for future research in this area are to directly examine the relationships between parent mindfulness/psychological flexibility and child and parent outcome measures (e.g., child pain or functional disability outcomes, child and parent socio-emotional well-being, etc.), the role of parent gender, as well as the potential mediating role of parent psychological flexibility between parent and child distress. It would also be important to offer mindfulness-based workshops to parents whose children have pain conditions, but whose children may not be involved in a group. Finally, designing and implementing a randomized control study, with an active control group is an important next step.

## Figures and Tables

**Table 1:** Content of Parent Mindfulness Workshop.

Schedule	Content
5 min	Name tags, tea/coffee, and snacks
10 min	**Welcome and Introduction to Mindfulness**
	*Teaching:* What is mindfulness?
	Presentation of Jon Kabat Zinn’s definition of mindfulness (Kabat-Zinn, 1996)
	Mindfulness gives us flexibility/choice in how we respond to situations in our lives (our children, our colleagues, our partners, our own internal experiences).
20 min	**Ice Breaker and Group Guidelines**
	*Activity 1:* Participants divide into dyads and are encouraged to pair up with someone unfamiliar. They are asked to introduce themselves to their partner and share elements in their lives that bring them joy. They are instructed to do so mindfully. The person speaking is asked to mindfully be aware of any sensations, emotions, thoughts, and/or judgments while they speak, and the listener is instructed to mindfully listen to their partner and be aware of any thoughts, emotions, sensations, or judgments that might take them away from mindful listening. After 4 min, participants are asked to switch roles and then introduce their partner to the group. The exercise is then processed by asking participants what they noticed during the activity and relating their observations back to mindfulness concepts (e.g., nonjudgment, present moment awareness, monkey mind, beginners mind).
	Review of group guidelines: (1) confidentiality, (2) respectful attitude
	The talking stick is introduced. When a participant is holding the talking stick, it is an invitation for them to speak from the heart and for others to give the speaker their full attention.
	*Activity 2:*
	*Teaching:* Suffering is optional (it is the distress, whether emotional or cognitive, associated with the pain that leads to a significant proportion of suffering)
	*Exercises:* Suffering Cup (participants add water to the cup to reflect the amount of space taken up by each of the following in their day-to-day lives: their child’s day-to-day physical symptoms, the parent’s own thoughts about their child’s symptoms, the parent’s own emotions about their child’s symptoms, the impact on the parent’s wellness of their child’s symptoms. Not only does their child’s actual symptoms lead to suffering, but a parent’s emotions and thoughts add to suffering, and are optional).
30 min	**Meditation: Mindful Awareness Meditation**
	Participants are taken through a mindful awareness practice of being aware of the quality of their thoughts, emotions, physical sensations, and sounds in the room, and approaching these with nonjudgmental curiosity and openness.
30 min	**Parenting According to Your Values**
	*Teaching:* Our values are a compass to guide interactions with our children. Mindfulness practice can help us connect with our values during difficult parenting situations.
	Parents are provided with a list of values and identify their top three values that guide their parenting.
	Parents were then asked to reflect on a situation where (a) they followed their values as a parent, and (b) they did not follow their values as a parent. In each situation, they notice their experience (their thoughts, emotions, and judgments).
	**Responding versus Reacting**
	*Teaching:* Responding versus reacting to strong sensations, emotions, or thoughts
15 min	How to bring mindfulness into your life
	Informal versus formal meditation practices
	List of local mindfulness resources and books/apps provided
	Home practice: To practice informal mindfulness
15 min	Stone ceremony
	Participants provide a good wish to one another, in turn.

Copyright: Danielle Ruskin, Ph.D., CPsych.

**Table 2:** Demographic Characteristics (*n* = 34 parents).

Characteristic	Full Sample	Chronic Pain	IBD
	(*n* = 34 Parents)	(*n* = 14 Parents)	(*n* = 20 Parents)
Parent gender, *n* (%)			
Female	27 (79%)	11 (79%)	16 (80%)
Male	7 (21%)	3 (21%)	4 (20%)
Adolescent age (years, mean SD)	14.9 ± 1.5	15.5 ± 1.6	14.2 ± 1.2 *
Adolescent gender, *n* (%)			
Female	23 (68%)	14 (100%)	9 (45%)
Male	11 (32%)	0 (0%)	11 (55%) *
Primary diagnosis for adolescent, *n* (%)	Musculoskeletal: 7 (50%)		Ulcerative colitis: 7 (41%)
	Neuropathic: 2 (14%)		
	Musculoskeletal + Neuropathic: 3 (21%)		Crohn’s disease: 10 (59%)
	Other ^1^: 2 (14%)		
Chronic pain: Adolescent’s duration of pain (months, mean SD)		59.7 ± 57.8	
IBD: Adolescent disease activity ^2^			13.1 ± 11.8

^1^ Other chronic pain includes headache and pelvic pain ^2^ Disease activity measured using the Self-Reported Disease Activity Questionnaire for IBD. Scores range from 0–85. Remission = <10; Mild disease activity = 10–30; Moderate disease activity = 35–60; Severe disease activity = 65+; IBD = Inflammatory Bowel Disease; * Significant difference between the chronic pain and IBD groups.

**Table 3:** Major Themes from the Post Session Questionnaire. MBSR: mindfulness-based stress reduction, STOP: Stop, Take a breath, Observe, Proceed.

Theme	Parents (*N* = 32)	Subthemes	Exemplar Quotes
Mindfulness Skills	29 (91%)	Present-Moment Awareness	“Trying to be more present and not just making it through the day” (Participant, IBD Group)
		Compassion	“I need to learn how to take care of myself so I can look after my child” (Participant, Chronic Pain Group)
		Acceptance	“The pain will stay, but we need to work collectively to develop and maintain coping strategies and work with our daughter to achieve some level of normalcy” (Participant, Chronic Pain Group)
		Secondary Suffering	“Understanding the potential for how much of my thinking can be consumed by worry for my child” (Participant, IBD Group)
		Importance of Regular Practice	“Strive to do mindfulness (MBSR) each day; to incorporate it into my daily routine” (Participant, IBD Group)
Not Alone	19 (59%)	Sense of Community/Shared Experience	“We aren’t alone—many people/families are dealing with pain issues, and that there are people and resources available to help” (Participant, Chronic Pain Group)
		Connection	“Parent sharing was very helpful” (Participant, IBD Group)
Psychological Flexibility	11 (34%)	Emotion Regulation	“The STOP method of responding—it’s a good quick way to remind myself to take a step back from a knee-jerk response when I’m on edge” (Participant, Chronic Pain Group)
		Perspective Taking	“Good experience to listen to other parents comments and struggles. Gain different perspectives and ideas” (Participant, IBD Group)
Parent–Child Interactions	8 (25%)	N/A	“Understanding how my actions can impact my daughter’s health” (Participant, IBD Group)
Self-Efficacy	7 (22%)	N/A	“I am not doing a bad job” (Participant, IBD Group)
Optimism/Positivity/Hope	6 (19%)	N/A	“There are people and resources available to help” (Participant, Chronic Pain Group)
Awareness of Values	5 (16%)	N/A	“To take the time to really realize what is important in the moment” (Participant, IBD Group)
